# Human Protein Subcellular Localization with Integrated Source and Multi-label Ensemble Classifier

**DOI:** 10.1038/srep28087

**Published:** 2016-06-21

**Authors:** Xiaotong Guo, Fulin Liu, Ying Ju, Zhen Wang, Chunyu Wang

**Affiliations:** 1 School of Instrumentation Science and Opto-electronics Engineering, Beihang University, Beijing, China; 2 School of Information Science and Technology, Xiamen University, Xiamen, China; 3 School of Computer Science and Technology, Harbin Institute of Technology, Harbin, China

## Abstract

Predicting protein subcellular location is necessary for understanding cell function. Several machine learning methods have been developed for computational prediction of primary protein sequences because wet experiments are costly and time consuming. However, two problems still exist in state-of-the-art methods. First, several proteins appear in different subcellular structures simultaneously, whereas current methods only predict one protein sequence in one subcellular structure. Second, most software tools are trained with obsolete data and the latest new databases are missed. We proposed a novel multi-label classification algorithm to solve the first problem and integrated several latest databases to improve prediction performance. Experiments proved the effectiveness of the proposed method. The present study would facilitate research on cellular proteomics.

Cells are highly ordered structure and contain various subcellular compartments that ensure the normal operation of the entire cell. These subcellular structures include nuclei, mitochondria, endoplasmic reticulum, Golgi apparatus, cell membrane, and extracellular matrix. The biological function of cells is executed by its unique proteins. Protein synthesized on the ribosome must be transported to its corresponding subcellular structures to play a normal biological function. If protein subcellular localization does not correspond to its position, serious loss of function or disorder occurs in organisms. Researchers found the aberrant protein subcellular localization in some cell lesions (such as cancer cells)^1^. The subcellular location of proteins is an important attribute of proteins, which is useful in determining protein function, revealing the mechanism of molecular interaction, and understanding the complex physiological processes^2^. The subcellular location of proteins is of great significance to cell biology, proteomics, and drug design research^3^.

Using conventional biochemical research methods, such as cell separation method, electronic microscopy, and fluorescence microscopy, to predict protein subcellular localization is expensive, time consuming, and laborious^4^. In today’s post-genome era, large amounts of protein sequence provide raw materials for the development of biological information and a stage for machine learning methods’ application in the field of life scienc^5^.

The typical protein subcellular location system based on machine learning methods includes the following four basic steps: (1) establishment of protein data set, (2) protein sequence feature extraction, (3) design of multi-label classification algorithm, and (4) construction of Web server^6^.

Databases for protein subcellular location, include LOCATE^7^, PSORTdb^8^, Arabidopsis Subcellular DB^9^, Yeast Subcellular DB^10^, Plant-PLoc^11^, LOCtarget^12^, LOC3D^13^, DBSubloc^14^, and PA-GOSUB^15^. However, none of the current works on computational protein subcellular localization have integrated these sources. Only part of the protein sequences were employed for training in previous works. In this paper, we collected existing related data sets and integrated a complete data set.

Feature extraction is a key process in various protein classification problems. Feature vectors are sometimes called as fingerprints of proteins. The common features include Chou’s PseACC representation^16^, K-mer and K-ship frequencies^17^, Chen’s 188D composition and physicochemical characteristics^18^, Wei’s secondary structure features^19^,^20^, and PSSM matrix features^21^. Several web servers were also developed for feature extraction of protein primary sequence, including Pse-in-one^22^, Protrweb^23^, and PseAAC^24^.

Proper classifier can help to improve the prediction performance. Support vector machine (SVM), k-nearest neighbor (kNN), artificial neural network, random forest (RF)^25^, and ensemble learning^26^,^27^ are often employed for special peptide identification. However, subcellular localization of a protein in essence is a multi-label classification problem, which is different from methods for identifying cellular factors (multi classification learning). Recently, several multi-label classification methods have been employed for subcellular localization in different species, including human^28^,^29^, plant^30^, virus^31^,^32^, eukaryote^33^,^34^, animal^35^. Features were also extracted according to n-gram^36^, Chou’s PseAAC representation^37^, and gene ontology^38^. They all focused on the features construction. Only the basic multi-label strategies were employed. Most of their researches have transferred SVM to multi labels. We found that advanced ensemble multi-label learning techniques would further improve the performance.

## Material and Methods

### Integration of multiple protein subcellular localization sources

In this section, we reconstruct the training set for human protein subcellular localization study. The new data set has a richer source and we further reduce the redundancy with CD-HIT^39^. Meanwhile, we expand the size of data sets, which render the training set data more comprehensive and provide a more convincing database for the multi-label classification learning step. The training set reconstruction will be introduced from two aspects, namely, data sources and data processing. The new dataset contains mainly two sources, which are LOCATE^7^ and Hum-mPLoc 2.0^40^.

About 526 (480+43+3 = 526) protein sequences are recorded as multi-label sequences (no repeat), which have two or more types of subcellular sites (the number of sites P_1_ is greater than or equals to 1) D_M1_. The protein sequence distribution on each subcellular site is shown in Table 1.

The subcellular sites contained in the proteins in Hum-mPLoc 2.0 are scarce, but parts of the protein data contain three or four subcellular sites. Proteins are rich and varied. Therefore, certain superiority is shown in terms of protein function.

From the LOCATE database, we directly obtained the document human.xml of the original XML format about subcellular localization of human. The document accommodates abundant information about human proteins. Our goal is to obtain 64,637 human protein amino acid FASTA sequences and the subcellular sites (site number P_2_ is more than or equals to 1) of these sequences. After a rigorous data processing, we obtain the reference data set containing 6776 different protein sequences (no repeat) D_2_. The 6776 protein sequences are distributed in 37 subcellular structures and possess two subcellular locations at most. Among these sequences, 4066 have only one type of subcellular location, which belongs to the single marker sequence data set D_S2_. Approximately 2710 protein sequences have two subcellular locations (site number P_2_ equals to 1), which belong to the multiple marker sequence data set D_M2_. A total of 9486 (4066+2710*2 = 9486) protein sequences (proteins locative, a repetitive protein sequence) correspond to 37 subcellular locations. The protein sequence distribution on each subcellular site is shown in Table 2.

Results of data processing indicate an extremely rich types of proteins and subcellular sites in the LOCATE database. However, the number of protein sequences, which have multiple subcellular sites, is relatively small, especially those belonging to three or more types of subcellular sites. This finding indicates that the protein data in the LOCATE have problems in functional diversity. To compensate for the limitations in the LOCATE database and Shen’s basic data set, we combine two types of data and reconstruct basic data sets. By combining Tables 1 and 2, we conclude that the 14 types of subcellular sites in Hum-mPLoc 2.0 are contained entirely in 37 types of subcellular sites in the LOCATE database, which is conducive to our data set reconstruction.

In order to prove the necessary of multi-label classification in the protein subcellular localization, it is required to compare the performances of multi-label and single-label classifiers. However, multi-label dataset cannot be used for single-label classifiers. Therefore, the data sets of multi-label protein sequences and single-label protein sequences were reconstructed separately, but they both come from the sources mentioned in the above section. The reconstructed data set was D_RM_, and the single labeled data set was D_RS_. Therefore,


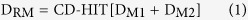



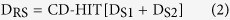



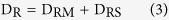


CD-HIT^39^ is a software for reducing the similarity of the protein sequences. It can delete the similar sequences from the data set. Here we made the similarity of each pair sequences is less than 40%. Table 3 shows the protein sequences of the reconstructed data set D_R_ and the subcellular sites.

### Features for subcellular localization

The above section mainly discusses a series of preprocessing with the data set. The reconstructed data set provides a reliable database for the study on the positioning method. This section focuses on specific features of protein subcellular localization based on machine learning.

In this section, three types of feature extraction methods are introduced based on the position-specific scoring matrix (PSSM)^41^, pseudo-amino acid composition^42^. In the long process of evolution, some characteristic genes are not eliminated but are selectively retained. These characteristics can effectively characterize the corresponding protein. Feature extraction methods based on PSSM are conducted to compare the protein sequence and rationally analyze with the invariance. PSSM matrix represents the comparison results between the input protein sequence and its homologous protein sequence in Swiss-Prot database. The multiple sequence alignment tools are HAlign^43^ and PSI-BLAST^44^ (position-specific initiated BLAST). Each input protein sequence generates a PSSM matrix after multiple sequence alignment. The elements in PSSM matrix characterize homology level between amino acids in some positions in the input protein sequence and the amino acid in the corresponding position in its homologous sequence. A smaller element value indicates higher conservation; lower conservation means that the amino acid in the position is prone to mutation. We extracted 20D and 420D features from the PSSM according to different parameters, which are described in detail in the supplementary materials.

The purpose of PseAAC is also to improve the accuracy of protein subcellular localization and the prediction of membrane protein. We extracted 188D features from PseACC, including 20D features of amino acid compositions, 24D features based on the contents of amino acids with certain physicochemical properties, 24D features of bivalent frequency and 120D features from eight physicochemical properties. It is described in detail in the supplementary materials, too.

### Multi-label classification ensemble learning method

We employed the ensemble multi-label classification method for improving the prediction performance. There have been no ensemble methods for multi-label classification in bioinformatics so far. Next we described the ensemble voting strategies of our method.

Basic classifiers are denoted as 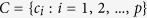, and the labels are denoted as 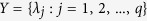.

#### MeanEnsemble algorithm

The prediction result is the probability that the sample is predicted to be 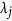 by 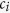. We calculate the average value of each column. Each training sample generates a set of q-dimensional vector:


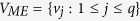


*v*_*j*_ is the probability that the sample belongs to the corresponding class label. If 0.5 ≤ *v*_*j*_ ≤ 1, the sequence belongs to *λ*_*j*_. If 0 ≤ *v*_*j*_ ≤ 0.5, the sequence does not belong to 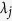.

#### MajorityVoteEnsemble algorithm

Every basic classifier separately predicted a sample. The prediction result is S, *S* ∈ (−1, +1). If S = −1, the sample is recognized as the counterexample by the base classifier; otherwise, it is identified as a positive example. We calculate the average value of each column, and each training sample generates a set of q-dimensional vector:


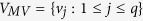


If *v*_*j*_ ≥ 0, the sample belongs to *λ*_*j*_; otherwise, it does not.

#### TopKEnsemble algorithm

In each column in the result matrix, P accuracy values are sorted in descending order and the average of the first K (K is determined by p) accuracy values is calculated to obtain a set of q-dimensional vector:


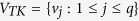


If 0.5 ≤ *v*_*j*_ <1, the sequence belongs to 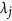. If 0 ≤ *v*_*j*_ <0.5, the sequence does not belong to 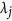.

**Figure 1:**
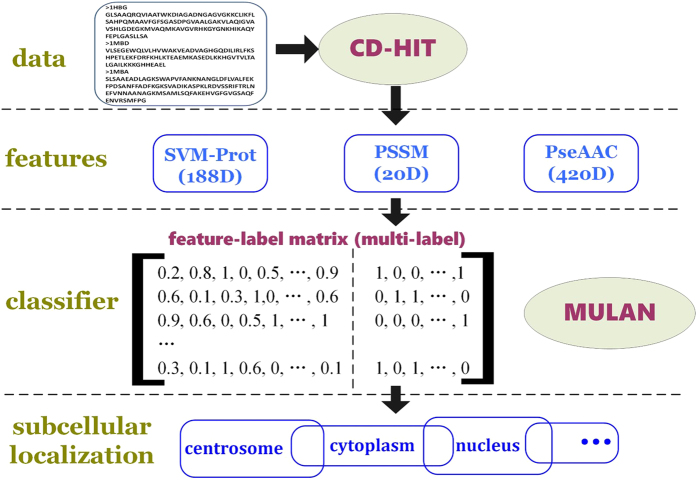
Working flow chart for our method.

The work flow of our protein subcellular localization prediction method can be shown in Fig. 1. In the data part, two sources of protein subcellular localization information were integrated. Then we tried three kinds of common features for representing the protein sequences. Multi-label classifier was employed for the prediction. The implementation was done with Mulan^45^, which is an open source machine learning software tool.

### Evaluation criteria and measurement

Average precision (AP)^46^: AP refers to the average accuracy of multi-label classification. This index is positively related to multi-label classification system performance. If AP = 1, the classification effect is the best. The calculation formula of AP is as follows:


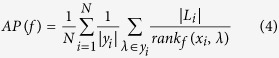



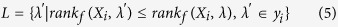


Here *N* is the number of all samples; |*y*_*i*_| is the number of the samples with label *y*_*i*_; *rank*(*x*_*i,*_*λ*) means the prediction value (sometimes viewed as probability) of sample *x*_*i*_ with label *λ*. We use AP as a primary measure of our comparative experiment.

## Results and Discussion

### Contrast experiments based on 188-dimensional classical features

**Figure 2:**
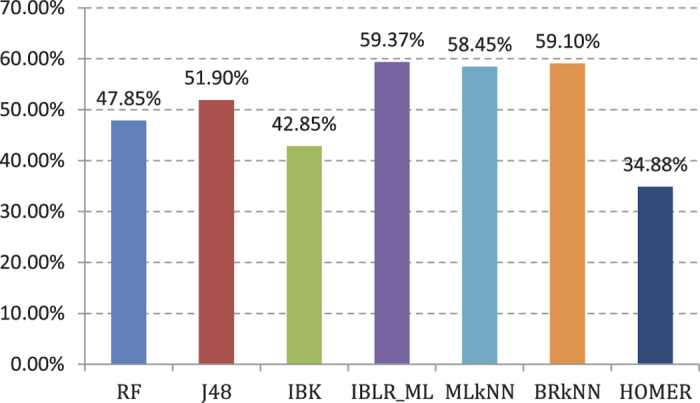
AP value comparison of 7 different basic classifiers in 188D features.

Experiment (1): Seven types of multi-labeled base classifiers are used to provide a fivefold cross validation for 188-dimensional feature^18^,^47^ training set. Classification performance is shown in Fig. 2. Detail value is shown in the Table S1 in supplementary materials. We take AP as the main reference indicator, and the AP values of the seven basic classifiers are shown in Fig. 2. The seven types of commonly used base classifiers in the experiment are random forest (RF), decision tree (J48), k nearest neighbor (IBK), logistic regression for multi-label classification (IBLR_ML)^48^, k nearest neighbor for multi-label classification (MLkNN)^49^, lazy multi-label classification (BRkNN)^50^, and Hierarchy of multi-label learners (HOMER)^51^. The former three classifiers are single-label ones, while the latter four are multi-label classifiers.

IBLR_ML achieves the highest AP value of the cross validation (59.37%), whereas HOMER has the lowest value (34.88%). The AP values of RF and IBK are less than 50%. We abandon the above three base classifiers with lower AP values. The four basic classifiers with higher AP values, namely, J48, IBLR_ML, MLkNN, and BRkNN, are integrated to the classification algorithm in Experiment (2).

**Figure 3:**
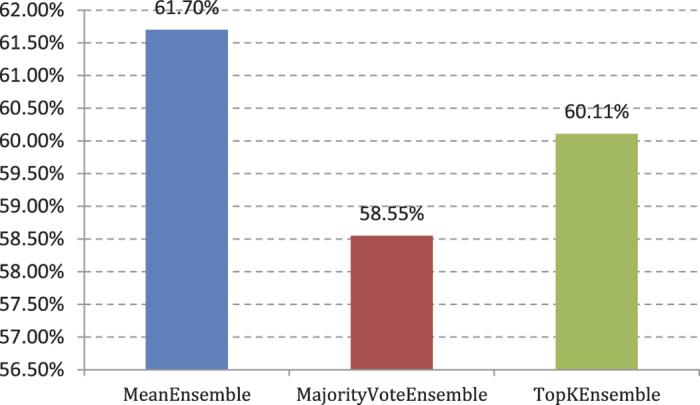
AP value comparison on 3 different ensemble classifier in 188D features.

Experiment (2): The four basic classifiers retained in Experiment (1) are integrated using our multi-label ensemble classification algorithms. We provide a fivefold cross validation for training sets. The AP values are shown in Fig. 3. Figure 3 demonstrates that the integration effect of MeanEnsemble multi-label ensemble classification algorithm for four types of base classifiers in Experiment (1) is optimal. The AP value is 61.70%.

The results of Experiments (1) and (2) show that the ensemble classification algorithm has a significant role in improving the accuracy of protein subcellular localization. We should notice that this is a serious imbalanced classification problem. The classifiers would prefer to the dominating labels. In the Table S4, we showed the detailed performances of individual subcellular locations. In the previous works, all the small classes were combined into a big class. We firstly tried to categorize 37 subcellular structures for prediction. Comparing with previous works, we have applied more subcellular structures and gotten more average accuracy.

### Contrast experiments based on PSSM-20-dimensional feature

**Figure 4:**
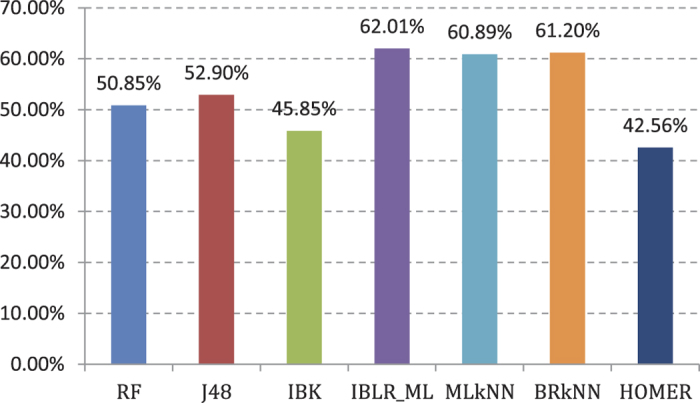
AP value comparison on 7 different basic multi-label classifiers in 20D features.

Experiment (3): Seven types of multi-labeled base classifiers are used to provide a fivefold cross validation for PSSM-20-dimensional feature training set. Classification performance is shown in Table S2 in the supplementary materials. Based on Table S2, we conclude that the AP value of fivefold cross validation that corresponds with PSSM-20d is better with better classification results. We still take AP as the main reference indicator, and the AP values of the seven base classifiers are shown in Fig. 4.

The chart shows that the IBLR_ML classifier obtains the highest AP value (62.01%). It has improved appropriately compared with the validation result of 188-dimensional feature training set. The rest of the base classifiers’ training effects have different degrees of improvement compared with Experiment (1). The four base classifiers with higher AP values, namely, J48, IBLR_ML, MLkNN, and BRkNN, are integrated to the classification algorithm in Experiment (4).

**Figure 5:**
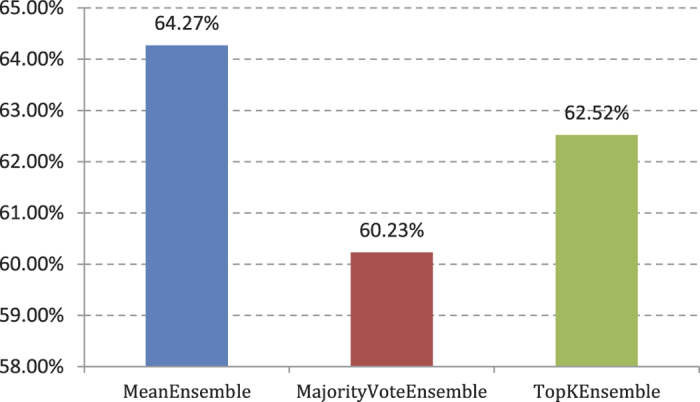
AP value comparison on 3 different ensemble multi-label classifiers in 20D features.

Experiment (4): We provide a fivefold cross validation for the training set with the same method as that in Experiment (2). The AP values are shown in Fig. 5.

The MeanEnsemble multi-label ensemble classification algorithm is still the best and better than the cross validation results of Experiment (2). The AP value reached 64.27%. TopKEnsemble and MajorityVoteEnsemble algorithms exhibit a larger increase compared with the training results in Experiment (2), but still less than the integrated effect of MeanEnsemble.

The results of Experiments (3) and (4) show that the ensemble classification algorithm has a significant role in improving the accuracy of protein subcellular localization again.

### Contrast experiments based on PseAAC-420-dimensional feature

**Figure 6:**
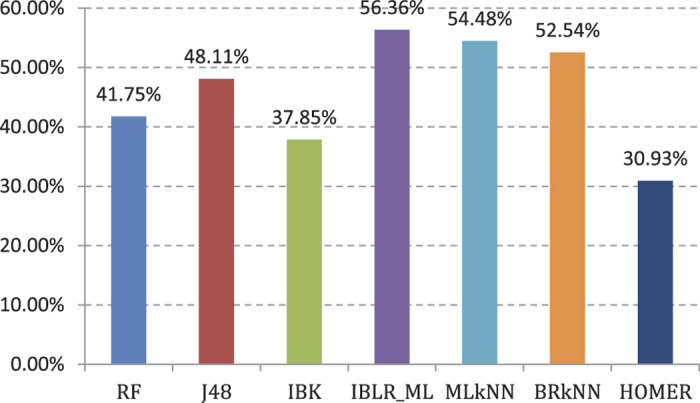
AP value comparison on 7 different basic multi-label classifiers in 420D features.

Experiment (5): Seven types of multi-labeled base classifiers are used to provide a fivefold cross validation for PseAAC-420-dimensional feature^42^ training set. Classification performance is shown in Table S3 in the supplementary materials. From Table S3 we can see that the AP values of fivefold cross validation that correspond with PseAAC-420d decline compared with 188d. The AP value of IBLR_ML is 56.36%, which is still the highest. It declines 3.01% and 5.65% compared with Experiments (1) and (3), respectively. The cross validation results are shown in Fig. 6.

The chart shows that the cross validation results of PseAAC-420-dimensional feature training set are the worst. The training results of the seven types of base classifiers decline compared with Experiments (1) and (3).

**Figure 7:**
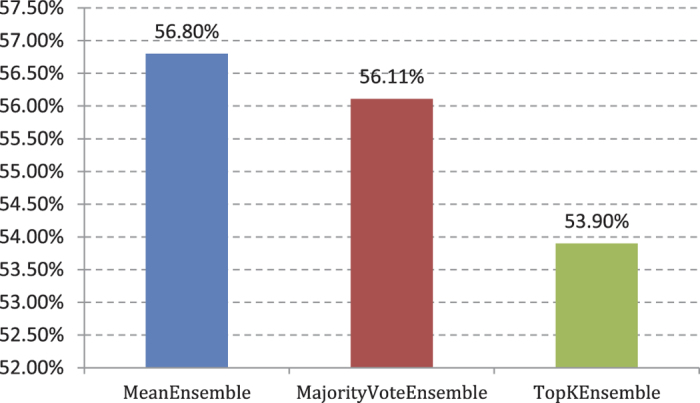
AP value comparison on 3 different ensemble multi-label classifiers in 420D features.

Experiment (4): We provide a fivefold cross validation for the training set with the same method as that in Experiment (4). The AP values are shown in Fig. 7.

### Comparison with state-of-the-art methods

In order to prove the performance of our method, we compared with the latest protein subcellular localization web servers, including IMMMLGP^28^, Hum-mPLoc 2.0^40^, mGOF-Loc^52^. The first one is a multi-label classifier, while the other two can only predict as single class. So we employ D_RM_ for the multi-label classification and D_RS_ for single-label classification. Since there are both multi-label and single-label classifiers, we cannot compare in the multi-label measurements, including Macro-averaged Precision, Micro-averaged Precision, Macro-averaged F-Measure, and Micro-averaged F-Measure. We just compare the average accuracy in the testing dataset. Table 4 showed the performance comparison in accuracy. From Table 4 we can see that our method outperformed the other latest methods. All of the accuracy rates come from 10-fold cross validation.

Besides that, we also tested our methods on other species, including plant, virus, eukaryote, and animal. Related datasets and performance were show in Table S5 and S6 in the supplementary materials. We concluded that our methods can also work on other species. But the performances were all poorer than human dataset. It is due to our integrated human protein subcellular localization dataset is more complete than other species. We will continue to collect the other species protein subcellular localization data in the future.

### Experiments analysis and discussion

We compare and analyze the training results of Experiments (1), (3), and (5) and Experiments (2), (4), and (6).

First, the seven cross validation results that correspond to PSSM-20-dimensional feature training set are better than the other two feature extraction algorithms. The IBLR_ML-based classifier shows the best performance, with the highest AP value of 62.01%. The contrast experimental results show that cross validation effects of PSSM-20 dimensional feature training set is the best for the base classifier.

Second, the cross validation results of MeanEnsemble, TopKEnsemble, and MajorityVoteEnsemble on PSSM-20-dimensional feature training set are higher than those of 188d and PseAAC-420d. The advantages of PSSM-20d in multi-label ensemble classification are shown.

By comparing the experimental results of the two groups, we conclude that the 20-dimensional feature extraction algorithm based on the PSSM is the most effective for protein subcellular localization.

Then we compare and analyze the training results of Experiments (3) and (4). Based on the integrated effect, the algorithm MeanEnsemble effect is the best, with an AP value of 64.27%, which is higher than predicting AP of any type of base classifier. The algorithm performance of MajorityVoteEnsemble is the worst, with an AP value fivefold cross training of only 60.23%. This value is lower than the multi-label classification results of the base classifiers IBLR_ML, BRkNN, and MLkNN with the same background data set, not embodying out the superiority of the integrated thought. It will be time consuming. By comparing the experimental results, we conclude that the multi-label classifier ensemble algorithm MeanEnsemble achieves the best effect for PSSM-20-dimensional feature training set. In the integrated four base classifiers, IBLR_ML shows the best multi-label learning performance.

## Conclusion

Protein subcellular localization with computational methods is a multi-label classification problem. State-of-the-art prediction methods employ traditional single label machine learning. We proposed novel multi-label ensemble classification techniques with novel hybrid protein features. Experiments proved the effectiveness of our features and the ensemble strategy. Several recent works have proved that ensemble learning^53^ and feature reduction^54^ can improve the performance of weak learning problems. However, the present work employed the simplest voting strategy and did not conduct any feature reduction techniques. Moreover, class imbalance occurred in protein subcellular localization problems. Imbalance learning for binary classification has been developed and applied in bioinformatics research^55^,^56^. However, no imbalance learning techniques exist for multi-class and multi-label classification. All these problems and application on large data^57^ would be investigated in future work.

## Additional Information

**How to cite this article**: Guo, X. *et al.* Human Protein Subcellular Localization with Integrated Source and Multi-label Ensemble Classifier. *Sci. Rep.* **6**, 28087; doi: 10.1038/srep28087 (2016).

## Supplementary Material

Supplementary Information

## Figures and Tables

**Table 1:** The protein sequences distribution on 14 subcellular sites.

Ordinal	Subcellular location	The number of proteins
1	centrosome	77
2	cytoplasm	817
3	cytoskeleton	79
4	endoplasmic reticulum	229
5	endosome	24
6	extracellular matrix	385
7	Golgi apparatus	161
8	lysosome	77
9	microsome	24
10	mitochondria	364
11	nucleus	1021
12	peroxisome	47
13	cell membrane	354
14	synaptic vesicle	22
Total number of non repeating protein sequences:		3106
Total number of protein Locative sequences:		3681
Multiple marker protein sequence data set D_M1_		526
Single marker protein sequence data set D_S1_		2580

**Table 2:** The protein sequences distribution on 37 subcellular sites in LOCATE.

Ordinal	Subcellular location	The number of proteins
1	Apical Plasma Membrane	44
2	Plasma Tube Basement Membrane	101
3	Cellular Component Unknown	6
4	Centrosome	27
5	Cytoplasm	1044
6	Cytoplasmic Vesicle	176
7	Cell Scaffold	72
8	Early Endosome	147
9	Endoplasmic Reticulum	343
10	Nuclear Body	454
11	ERGIC	7
12	Extracellular Matrix	394
13	Golgi Apparatus	387
14	Golgi Cis Cisterna	27
15	Golgi Trans Cisterna	11
16	Golgi Trans Face	27
17	Mitochondrial Inner Membrane	8
18	Late Endosomes	34
19	Lipid Lowering Granule	1
20	Lysosome	222
21	Medial-Golgi	17
22	Black Body	20
23	Microtubule Organizing Center	8
24	Micro Tube	4
25	Mitochondrion	279
26	Nuclear Membrane	155
27	Nucleolus	810
28	Nucleus	2721
29	Mitochondrial Outer Membrane	2
30	Peroxisome	128
31	Cell Membrane	1711
32	Muscle Fiber Membrane	5
33	Secretory Granules	25
34	Secretory Vesicle	8
35	Synaptic Vesicle	29
36	Tight Junction	26
37	Transport Vesicle	6
Total number of non repeating protein sequences:		6776
Total number of protein Locative sequences:		9486
Multiple marker protein sequence data set D_M2_		2710
Single marker protein sequence data set D_S2_		4066

**Table 3:** Subcellular sites and protein sequences distribution in D_R._

The multiple labeled set D_RM_			The single labeled set D_RS_		
Ordinal	Subcellular location	The number of proteins	Ordinal	Subcellular location	The number of proteins
1	Apical Plasma Membrane	16	1	centrosome	44
2	Plasma Tube Basement Membrane	29	2	cytoplasm	508
3	Cellular Component Unknown	4	3	cytoskeleton	46
4	Centrosome	37	4	endoplasmic reticulum	77
5	Cytoplasm	542	5	endosome	163
6	Cytoplasmic Vesicle	29	6	extracellular matrix	419
7	Cell Scaffold	43	7	Golgi apparatus	125
8	Early Endosome	52	8	lysosome	86
9	Endoplasmic Reticulum	43	9	Micro Tube	11
10	Nuclear Body	179	10	mitochondria	355
11	ERGIC	4	11	nucleus	952
12	Extracellular Matrix	68	12	peroxisome	56
13	Golgi Apparatus	147	13	cell membrane	565
14	Golgi Cis Cisterna	7	14	synaptic vesicle	16
15	Golgi Trans Cisterna	3	15	Cytoplasmic Vesicle	17
16	Golgi Trans Face	11	16	Black Body	4
17	Mitochondrial Inner Membrane	4	17	Nuclear Membrane	1
18	Late Endosomes	16	18	Secretory Granules	1
19	Lipid Lowering Granule	1	19	Secretory Vesicle	2
20	Lysosome	39	\	\	\
21	Medial-Golgi	7	\	\	\
22	Black Body	2	\	\	\
23	Microtubule Organizing Center	1	\	\	\
24	Micro Tube	15	\	\	\
25	Mitochondrion	52	\	\	\
26	Nuclear Membrane	46	\	\	\
27	Nucleolus	268	\	\	\
28	Nucleus	768	\	\	\
29	Mitochondrial Outer Membrane	1	\	\	\
30	Peroxisome	11	\	\	\
31	Cell Membrane	271	\	\	\
32	Muscle Fiber Membrane	1	\	\	\
33	Secretory Granules	9	\	\	\
34	Secretory Vesicle	3	\	\	\
35	Synaptic Vesicle	12	\	\	\
36	Tight Junction	9	\	\	\
37	Transport Vesicle	4	\	\	\
The reconstructed multiple labeled set D_RM_					1354
The reconstructed single labeled set D_RS_					3448
The reconstructed protein subcellular localization data set D_R_					4802

**Table 4:** Accuracy comparison with state-of-the-art methods.

Methods	Average Precision
IMMMLGP	0.5725
Hum-mPLoc 2.0	0.5644
mGOF-loc	0.582
Our method(188D features)	**0.5937**
Our method(20D features)	**0.6201**
