# Supplementary material for: Human Protein Subcellular Localization with Integrated Source and Multi-label Ensemble Classifier
Source: Sci Rep. 2016 Jun 21;6:28087. doi: 10.1038/srep28087 (PMC4914962; doi:10.1038/srep28087)
Supplement: Supplementary Information [file srep28087-s1.doc]

**Human Protein Subcellular Localization with Integrated Source and Multi-label Ensemble Classifier**

Xiaotong Guo1, Fulin Liu1, Ying Ju2, Zhen Wang2, Chunyu Wang3*

1. School of Instrumentation Science and Opto-electronics Engineering, Beihang University, Beijing, China
2. School of Information Science and Technology, Xiamen University, Xiamen, China
3. School of Computer Science and Technology, Harbin Institute of Technology, Harbin, China

Email address:

*corresponding author: Chunyu Wang, chunyu@hit.edu.cn

**Supplementary materials for “Human Protein Subcellular Localization with Integrated Source and Multi-label Ensemble Classifier”**

**1. Datasets in detail**

(1) Human protein sequences in the LOCATE database ([http://locate.imb.uq.edu.au](http://locate.imb.uq.edu.au/)). The subcellular sites of protein sequences are selected through experimental methods for high-throughput immunofluorescence labeling, or through manually checking from 1700 species of peer-reviewed publications, with high reliability. The LOCATE database contains two types of labeled protein subcellular sites: human protein and mouse protein sequences. A total of 64,637 human protein sequences are marked with 37 types of subcellular structure. The LOCATE database opens a free data download link, where the original file human.xml on human protein sequences can be downloaded.

(2) Hum-mPLoc 2.0. The protein sequences and subcellular sites come from the Swiss-Prot database (<http://www.ebi.ac.uk/swissprot/>). After a rigorous data processing, he obtained a reference data set that contained 3106 different protein sequences (no repeat) D_1_. These protein sequences are distributed in 14 subcellular structures. Among these protein sequences, 2580 have only one type of subcellular location, which belongs to the single marker sequence data set D_S1_. About 480 protein sequences have two subcellular locations, 43 protein sequences possess three subcellular locations, and 3 protein sequences possess four subcellular locations. A total of 3681 (2580+480*2+43*3+3*4=3681) protein sequences (proteins locative, a repetitive protein sequence) correspond to 14 subcellular locations.

The following is the principle of CD-HIT:

(1) All the sequences of the target data set are arranged in descending order according to length;

(2) The No. 1 sequence S_1_ is assigned as the first sequence φ_1_ and is compared with sequence S2. If the similarity between new sequences and representative sequences (maximum length) of the former sequences is greater than the cutoff, this sequence is placed in the former sequences, or it is assigned as a representative of a new type of sequences.

Some sequence similarities are likely to exist between protein sequences on the same site in two data sets. The actual prediction performance of the model is reduced although the accuracy of the model is improved, which influences the effect of multi-label classification to a large extent. Therefore, performing targeted CD-HIT redundancy processing is necessary for the combined multi-tag and single tag sets. This method can effectively eliminate similar sequences after merging and can protect the species diversity of the data set.

The sequence similarity ratio is set to 40%. The reconstructed data set is:

D_R_=(m, n)T.

‘m’ represents the number of protein sequences in the set, and ‘n’ indicates the number of subcellular sites. D_R_=(4802,37). The multiple labeled set D_RM_=(3448,37), and the single labeled set D_RS_=(1354,19). The number of subcellular sites in the D_RS_ collection is 19.

**2. Features in detail**

- 1. **Position-specific scoring matrix PSSM**

PSSM matrix represents the comparison results between the input protein sequence and its homologous protein sequence in Swiss-Prot database. The multiple sequence alignment tools are HAlign and PSI-BLAST (position-specific initiated BLAST). Each input protein sequence generates a PSSM matrix after multiple sequence alignment. The elements in PSSM matrix characterize homology level between amino acids in some positions in the input protein sequence and the amino acid in the corresponding position in its homologous sequence. A smaller element value indicates higher conservation; lower conservation means that the amino acid in the position is prone to mutation.

PSSM is an L × 20 matrix:

20 (column): 20 types of amino acids that make up the protein; j column represents the amino acid A_j_;

L (line): the length of the tested protein, that is, the number of amino acids. The i line represents the amino acid in the i position in the tested protein sequence;

The element φ (i, j) represents the possibility score of amino acid A_j_’s appearance on the i position in the tested sequence.

The PSSM matrix is shown as follows:

.

- 1. **A 20-dimensional feature extraction algorithm based on PSSM**

We first obtain the PSSM matrix as follows:

(1) We select the latest version of the Swiss-Prot as the background alignment database, which contains 548,758 protein sequences. We also choose BLAST+ as multiple sequence alignment tool;

(2) We format the Swiss-Prot database with the BLAST+ command;

(3) We choose a single protein sequence as a unit cutting data set. The data set is divided into a plurality of small files that contain single protein sequences;

(4) We input file of a single protein one by one and compare input files with the format of the Swiss-Prot database with the PSI-BLAST tool. At the end of the comparison, we obtain the PSSM matrix of each protein file.

Because BLAST+ can only compare one input sequence and extract one PSSM matrix, the PSI-BLAST multiple sequence alignment is a batch process.

The main significance of PSSM matrix is the possibility that the amino acid on a position in the tested protein sequences is replaced by other amino acids in the evolution process. Thus, we make each type of amino acid (a total of 20) as a unit and calculate the average score of this amino acid being substituted. Each tested protein sequence generates a group of 20-dimensional feature vector, on behalf of the average score of 20 amino acids being substituted in the sequence, as shown in the following formula:

$$V_{P}=[\overline{E_{1}},\overline{E_{2}},...,\overline{E_{20}}]^{T}$$

.

$\overline{E_{j}}$ is the average probability of all the original amino acids in a tested protein sequence being replaced by the amino acid Aj.

**2.3 PseAAC (pseudo-amino acid composition) feature extraction algorithm**

The purpose of PseAAC is to improve the accuracy of protein subcellular localization and the prediction of membrane protein. After feature extraction, the transformed vector can be divided into two parts, namely, the contents of the 20 amino acids in the sequence:$(v_{1},v_{2},v_{3}...v_{20})^{T}=(\frac{n_{1}}{L},\frac{n_{2}}{L},\frac{n_{3}}{L}...\frac{n_{20}}{L})$ and λ dimensional feature vector, which reveals the effect of residue in protein sequences on the physical and chemical properties of protein.

The first part of the feature vector is V_AA_:

$V_{AA}=[v_{1},v_{2},...,v_{20}]^{T}$.

The next formula characterizes the frequency of the 20 amino acids in the protein sequence (the content composition). The second part of the feature vector is V_c_:

$V_{C}=[\delta_{1},\delta_{2},...,\delta_{\lambda}]^{T}$.

The feature vector extracted by the entire PseAAC algorithm is V_PseAAC_, and then the V_PseAAC_ is the orthogonal sum of V_AA_ and Vc after normalization:

$$V_{PseAAC}=V_{AA}\cdot V_{C}=[v_{1},v_{2},...,v_{20},\delta_{1},\delta_{2},...,\delta_{\lambda}]^{T}$$

$v_{i}=\frac{v_{i}}{\sum_{i=1}^{20} v_{i}+w\sum_{j=1}^{\lambda} \delta_{\lambda}}$, $1\leq i\leq20$

$\delta_{j}=\frac{w\delta_{i}}{\sum_{i=1}^{20} f_{i}+w\sum_{j=1}^{\lambda} \delta_{\lambda}}$,$1\leq j\leq\lambda$.

In the above formula, *w* is the weight factor. $\delta_{\lambda}$ is the correlation factor of the protein sequence with the most adjacent λ amino acid residues:

$$\delta_{\lambda}=\frac{1}{L-\lambda}\sum_{i=1}^{L-\lambda} J_{i,\lambda},(\lambda<L)$$

.

is the *q* function of the $R_{\lambda}$ amino acid, and Г is the total number of functions considered.

**2.4 Algorithm based on AA composition**

The algorithm based on AA composition has been previously formulated. By calculating the frequencies of 20 amino acids in the protein sequence and using these frequencies to represent a specific protein sequence, each sequence becomes a 20d vector after features conversion:

$(v_{1},v_{2},v_{3}...v_{20})^{T}=(\frac{n_{1}}{L},\frac{n_{2}}{L},\frac{n_{3}}{L}...\frac{n_{20}}{L})$ (2-1)

where $n_{i}$ $(i=1,2,3...20)$ represents the quantity of an AA in the protein sequence. Obviously, $\sum_{i=1}^{20} v_{i}=1$.

**2.5 Algorithm based on the distribution of AAs with certain physicochemical properties**

The nature of AAs is determined by their side chains, and these side chains vary in shape, charge, and hydrophobicity. AAs sequences thus have different structural features and physiological functions. Based on this perspective, we employed eight physicochemical properties of AAs such as SS, solvent accessibility, normalized Van der Waals volume, hydrophobicity, change, polarizability, polarity, and surface tension.

We calculated the characteristic value of the distribution of AAs with certain physicochemical properties (D). Using SS as an example:

To the AAs of EALMQKRH group, making the position of the first, 25%, 50%, 75%, and 100% of AAs chain represented by $p_{11}$,$p_{12}$…$p_{15}$, respectively, and the lengths from $p_{11}$,$p_{12}$…$p_{15}$ to the head of this protein sequence are $DSS_{11},DSS_{12}...DSS_{15}$, respectively. We can calculate similar parameters of two other AA SS as $DSS_{21},DSS_{22}...DSS_{25}$,$DSS_{31},DSS_{32}...DSS_{35}$. $V_{1},V_{2}...V_{15}$ can then be represented as:

$\left[\begin{matrix} V_{1} & V_{6} & V_{11} \\ V_{2} & V_{7} & V_{12} \\ V_{3} & V_{8} & V_{13} \\ V_{4} & V_{9} & V_{14} \\ V_{5} & V_{10} & V_{15} \end{matrix}\right]=\left[\begin{matrix} \frac{DSS_{11}}{L} & \frac{DSS_{21}}{L} & \frac{DSS_{31}}{L} \\ \frac{DSS_{12}}{L} & \frac{DSS_{22}}{L} & \frac{DSS_{32}}{L} \\ \frac{DSS_{13}}{L} & \frac{DSS_{23}}{L} & \frac{DSS_{33}}{L} \\ \frac{DSS_{14}}{L} & \frac{DSS_{24}}{L} & \frac{DSS_{34}}{L} \\ \frac{DSS_{15}}{L} & \frac{DSS_{25}}{L} & \frac{DSS_{35}}{L} \end{matrix}\right]$ (2-2)

Thus, 15d feature vectors may be extracted from the SS property. We can extract 120d feature vectors after the eight physicochemical properties are analyzed.

In 2003, Cai established a method of features extraction based on the composition and distribution of amino acids combined with their physicochemical properties. A total of 188d features were extracted, including the 120d features we used in this paper (2-2), 20d features of AA compositions (2-1), 24d features based on the contents of AAs with certain physicochemical properties (2-3), and 24d features of bivalent frequency (2-4) based on the eight physicochemical properties described above. We will demonstrate that the effectiveness of our 120d features is superior to that of the 188d combined features through multiple sets of experiments.

(2-3)

(2-4)

**3. Multi-label classifier in detail**

**3.1 Multi-label classification learning**

Compared with the single label classification learning problems, all samples in the multi-label classification learning can have one or more class labels. A single example label is no longer unique, which means that the label is no longer exclusive. The objective of multi-label learning is to give the unknown sample to all appropriate labels. The learning process can be described as follows:

Characteristic properties of samples:

$$X=\{x_{i}:i=1,2,...,m\}$$

Finite labeled set:

$$Y=\{\lambda_{j}:j=1,2,...,q\}$$

Given learning sample set:

$$S=\{(x_{i},L_{i}):i=1,2,...,k\}$$

$x_{i}\in X,L_{i}\subseteq Y$; constructing a classifier C is required, which can be set to make an effective mark for unknown samples:

$T=\{(x_{k+i},?):i=1,2,...,p\}$.

Two main approaches to solve multi-label classification problems are as follows:

(1) Algorithm independency. Through the decomposition of the sample sets, a multi-label learning problem is transformed into multiple binary classification or multi-class classification (single marked) learning problem to deal with, and integrate each single label classification result as a multi-label learning result. The importance of this algorithm is still the ordinary single label classification that has no relationship with multi-label learning algorithm.

(2) Algorithm dependency, which is based on the single label classification algorithm, can be effectively extended and reconstructed to deal with multi-label classification problem. The common algorithms are Rank-SVM and ML-KNN.

**3.2 Mulan**

Mulan uses Weka for multi-label classification. Therefore, Mulan also supports arff file format. The difference between the formats is the addition of some attributes on the foundation of the original arff file content; a label is added at the end of each necessary feature value sequence in the original arff files. The label value can only be 0 or 1. A comma exists in the English format between each label value. The label value is represented if a label exists (whether it belongs to the label corresponding to the category) in the feature value sequence. 0 indicates absence, and 1 indicates presence.

The ARFF file contains three types of feature values and five types of labels. Feature values in the table represent the samples with two types of labels, namely, label2 and label3. Element order in the (0,1) set sequence corresponds with the labels’ order in the file header. With the two files above, we can use the Mulan to train the data set.

Mulan not only provides a set of data training function, but also provides validation and prediction function for the test set to train models and then predict with models. In a multi-label test, only the test set’s feature arff files are needed. The XML file is not necessary. The format of the test set’s feature arff file should be the same with those of the training set, that is, label sequence should be added at the end of each feature value sequence. The label values can all be "1," "0," and "?."

**4. Experiments in detail**

**4.1 Metric values for different classifiers**

It has been showed in Fig 1-3 in the paper. Table S1-S3 have listed the detailed values for different metric.

**Table S1** 5-fold cross validation on 7 kinds of multi labeled base classifiers(188d)

| evaluating indicators  base classifiers | Average Precision | Macro-averaged  Precision | Micro-averaged  Precision | Macro-averaged  F-Measure | Micro-averaged  F-Measure |
| --- | --- | --- | --- | --- | --- |
| RF | 0.4785±0.0129 | 0.2648±0.0139 | 0.5951±0.0285 | 0.2901±0.0063 | 0.1768±0.0222 |
| J48 | 0.5190±0.0031 | 0.2273±0.0250 | 0.4571±0.0071 | 0.3290±0.0034 | 0.1979±0.0219 |
| IBK | 0.4285±0.0079 | 0.2480±0.0411 | 0.5495±0.0261 | 0.3070±0.0086 | 0.1789±0.0256 |
| IBLR_ML | 0.5937±0.0088 | 0.1465±0.0162 | 0.5633±0.0194 | 0.2953±0.0061 | 0.0910±0.0170 |
| MLkNN | 0.5845±0.0105 | 0.2032±0.0239 | 0.6399±0.0224 | 0.2455±0.0185 | 0.1677±0.0262 |
| BRkNN | 0.5910±0.0109 | 0.2193±0.0330 | 0.6806±0.0356 | 0.1686±0.0118 | 0.1524±0.0223 |
| HOMER | 0.3488±0.0035 | 0.1886±0.0141 | 0.3456±0.0133 | 0.3263±0.0066 | 0.1825±0.0164 |

**Table S2** 5-fold cross validation on 7 kinds of multi labeled base classifiers (PSSM-20d)

| Evaluating indicators  Base classifiers | Average Precision | Macro-averaged  Precision | Micro-averaged  Precision | Macro-averaged  F-Measure | Micro-averaged  F-Measure |
| --- | --- | --- | --- | --- | --- |
| RF | 0.5085±0.0029 | 0.2584±0.0112 | 0.6012±0.0321 | 0.3010±0.0100 | 0.1800±0.0300 |
| J48 | 0.5290±0.0040 | 0.2312±0.0265 | 0.4633±0.0088 | 0.3302±0.0029 | 0.1896±0.0310 |
| IBK | 0.4585±0.0080 | 0.2520±0.0369 | 0.5285±0.0303 | 0.3200±0.0094 | 0.1821±0.0243 |
| IBLR_ML | 0.6201±0.0056 | 0.1428±0.0201 | 0.5729±0.0200 | 0.3053±0.0074 | 0.0901±0.0160 |
| MLkNN | 0.6089±0.0111 | 0.2130±0.0421 | 0.6500±0.0341 | 0.2510±0.0201 | 0.1597±0.0259 |
| BRkNN | 0.6120±0.0102 | 0.2078±0.0239 | 0.7026±0.0420 | 0.1756±0.0123 | 0.1589±0.0244 |
| HOMER | 0.4256±0.0029 | 0.1795±0.0210 | 0.3323±0.0141 | 0.3099±0.0072 | 0.1999±0.0159 |

**Table S3** 5-fold cross validation on 7 kinds of multi labeled base classifiers (PseAAC-420d)

| Evaluating indicators  base classifiers | Average Precision | Macro-averaged  Precision | Micro-averaged  Precision | Macro-averaged  F-Measure | Micro-averaged  F-Measure |
| --- | --- | --- | --- | --- | --- |
| RF | 0.4175±0.0061 | 0.2568±0.0144 | 0.5748±0.0325 | 0.2141±0.0078 | 0.1545±0.0124 |
| J48 | 0.4811±0.0094 | 0.2224±0.0236 | 0.4008±0.0084 | 0.2902±0.0046 | 0.1894±0.0194 |
| IBK | 0.3788±0.0125 | 0.2074±0.0159 | 0.4672±0.0085 | 0.2763±0.0081 | 0.1617±0.0227 |
| IBLR_ML | 0.5636±0.0111 | 0.1481±0.0288 | 0.5412±0.0155 | 0.2332±0.0081 | 0.1137±0.0262 |
| MLkNN | 0.5448±0.0036 | 0.1755±0.0179 | 0.6019±0.0269 | 0.1517±0.0115 | 0.1485±0.0214 |
| BRkNN | 0.5254±0.0126 | 0.1511±0.0219 | 0.5880±0.0454 | 0.1843±0.0432 | 0.1476±0.0236 |
| HOMER | 0.3093±0.0033 | 0.1597±0.0084 | 0.2536±0.0029 | 0.2569±0.0041 | 0.1599±0.0074 |

**4.2 Performance on each subcellular localization**

We list the performance on each subcellular localization with 10-fold cross validation on 188D features and MLkNN in Table S4

**Table S4** Performance on each subcellular localization with 10-fold cross validation on 188D features and MLkNN

|  | **Macro-averaged Precision** | **Macro-averaged Recall** | **Macro-averaged F-Measure** |
| --- | --- | --- | --- |
| **Lipid_Particles** | 0.9000±0.1890 | 0.9000±0.1890 | 0.9000±0.1890 |
| **Extracellular** | 0.6423±0.0621 | 0.1153±0.0129 | 0.1869±0.0205 |
| **Early_Endosomes** | 0.0000±0.0000 | 0.0000±0.0000 | 0.0000±0.0000 |
| **Endoplasmic_Reticulum** | 0.0000±0.0000 | 0.0000±0.0000 | 0.0000±0.0000 |
| **Nuclear_Envelope** | 0.0000±0.0000 | 0.0000±0.0000 | 0.0000±0.0000 |
| **Mitochondria** | 0.6273±0.1096 | 0.1760±0.0254 | 0.2670±0.0404 |
| **Cytoplasmic_Vesicles** | 0.0000±0.0000 | 0.0000±0.0000 | 0.0000±0.0000 |
| **Centrosome** | 0.0000±0.0000 | 0.0000±0.0000 | 0.0000±0.0000 |
| **Endosomes** | 0.0000±0.0000 | 0.0000±0.0000 | 0.0000±0.0000 |
| **Cellular_Component_Unknown** | 1.0000±0.1000 | 1.0000±0.1000 | 1.0000±0.1000 |
| **Golgi_Cis_Cisterna** | 0.5000±0.2250 | 0.5000±0.2250 | 0.5000±0.2250 |
| **Cytoskeleton** | 0.0000±0.0000 | 0.0000±0.0000 | 0.0000±0.0000 |
| **Transport_Vesicle** | 0.6000±0.2040 | 0.6000±0.2040 | 0.6000±0.2040 |
| **Microtubule** | 0.0000±0.0000 | 0.0000±0.0000 | 0.0000±0.0000 |
| **Peroxisome** | 0.0000±0.0000 | 0.0000±0.0000 | 0.0000±0.0000 |
| **Cytoplasm** | 0.0500±0.0223 | 0.0017±0.0000 | 0.0033±0.0001 |
| **Outer_Mitochondrial_Membrane** | 0.9000±0.1890 | 0.9000±0.1890 | 0.9000±0.1890 |
| **Nucleolus** | 0.0000±0.0000 | 0.0000±0.0000 | 0.0000±0.0000 |
| **Apical_Plasma_Membrane** | 0.1000±0.0890 | 0.1000±0.0890 | 0.1000±0.0890 |
| **Melanosome** | 0.4000±0.3040 | 0.4000±0.3040 | 0.4000±0.3040 |
| **Late_Endosomes** | 0.2000±0.1560 | 0.2000±0.1560 | 0.2000±0.1560 |
| **Golgi_Trans_Face** | 0.3000±0.2010 | 0.3000±0.2010 | 0.3000±0.2010 |
| **Secretory_Vesicles** | 0.6000±0.2040 | 0.6000±0.2040 | 0.6000±0.2040 |
| **Golgi_Trans_Cisterna** | 0.7000±0.3010 | 0.7000±0.3010 | 0.7000±0.3010 |
| **Plasma_Membrane** | 0.6081±0.0909 | 0.1836±0.0320 | 0.2795±0.0480 |
| **Tight_Junction** | 0.3000±0.2010 | 0.3000±0.2010 | 0.3000±0.2010 |
| **Medial-Golgi** | 0.5000±0.3250 | 0.5000±0.3250 | 0.5000±0.3250 |
| **ERGIC** | 0.7000±0.1610 | 0.7000±0.1610 | 0.7000±0.1610 |
| **Microtubule_Organizing_Center** | 0.9000±0.1890 | 0.9000±0.1890 | 0.9000±0.1890 |
| **Inner_Mitochondrial_Membrane** | 0.7000±0.3010 | 0.7000±0.3010 | 0.7000±0.3010 |
| **Secretory_Granule** | 0.2000±0.1560 | 0.2000±0.1560 | 0.2000±0.1560 |
| **Sarcolemma** | 0.9000±0.1890 | 0.9000±0.1890 | 0.9000±0.1890 |
| **Golgi_Apparatus** | 0.0000±0.0000 | 0.0000±0.0000 | 0.0000±0.0000 |
| **Basolateral_Plasma_Membrane** | 0.1000±0.0890 | 0.1000±0.0890 | 0.1000±0.0890 |
| **Synaptic_Vesicles** | 0.0000±0.0000 | 0.0000±0.0000 | 0.0000±0.0000 |
| **Lysosomes** | 0.0000±0.0000 | 0.0000±0.0000 | 0.0000±0.0000 |
| **Nucleus** | 0.6570±0.0641 | 0.4350±0.0444 | 0.5199±0.0507 |

**4.3 Performance on other species**

In our work, we integrate the human protein sequences and subcellular localization information. Our features and multi-label classifier seemed to work well. However, can it perform well on other species? We tried our method on plant, virus, eukaryote, animal. The datasets come from <http://www.jci-bioinfo.cn/>, as shown in Table S5. The performance on different features and multi-label classifier (IBLR_ML) were listed in Table S6. They come from 5-cross validation in the training sets. From Table S6 we can see that our methods can also work on other species. But the performances were all poorer than human dataset. It is due to our integrated human protein subcellular localization dataset is more complete than other species. We will continue to collect the other species protein subcellular localization data in the future.

**Table S5** Data sources for other species

| Datasets | URL |
| --- | --- |
| plant | <http://www.jci-bioinfo.cn/iLoc-Plant> |
| virus | <http://www.jci-bioinfo.cn/iLoc-Virus> |
| eukaryote | <http://www.jci-bioinfo.cn/iLoc-Euk> |
| animal | <http://www.jci-bioinfo.cn/iLoc-Animal> |

**Table S6** Average Precision with different features on different species

| Datasets | 188D | 20D | 420D |
| --- | --- | --- | --- |
| plant | 0.4968±0.0096 | 0.5233±0.0112 | 0.4231±0.0336 |
| virus | 0.3266±0.0085 | 0.3662±0.0129 | 0.3001±0.0291 |
| eukaryote | 0.4646±0.0091 | 0.4921±0.0132 | 0.4469±0.0285 |
| animal | 0.5784±0.0156 | 0.5977±0.0176 | 0.5287±0.0236 |
